# The Regulation and Functions of Endogenous Retrovirus in Embryo Development and Stem Cell Differentiation

**DOI:** 10.1155/2021/6660936

**Published:** 2021-02-27

**Authors:** Yangquan Xiang, Hongqing Liang

**Affiliations:** Division of Human Reproduction and Developmental Genetics, Women's Hospital, and Institute of Genetics, Zhejiang University School of Medicine, Hangzhou, Zhejiang, China

## Abstract

Endogenous retroviruses (ERVs) are repetitive sequences in the genome, belonging to the retrotransposon family. During the course of life, ERVs are associated with multiple aspects of chromatin and transcriptional regulation in development and pathological conditions. In mammalian embryos, ERVs are extensively activated in early embryo development, but with a highly restricted spatial-temporal pattern; and they are drastically silenced during differentiation with exceptions in extraembryonic tissue and germlines. The dynamic activation pattern of ERVs raises questions about how ERVs are regulated in the life cycle and whether they are functionally important to cell fate decision during early embryo and somatic cell development. Therefore, in this review, we focus on the pieces of evidence demonstrating regulations and functions of ERVs during stem cell differentiation, which suggests that ERV activation is not a passive result of cell fate transition but the active epigenetic and transcriptional regulation during mammalian development and stem cell differentiation.

## 1. Introduction

ERVs belong to a Class family of retrotransposon elements in the genome. Together with DNA transposons, they are known as transposable elements (TEs), which are derived from DNA fragments able to transpose within the genome. Due to their capacities to hop around and copy themselves in the genome, TEs are considered one of the main driving forces in reconstructing the genome during mammalian evolution. To date, TEs have mostly lost the ability to transpose [1, 2], considering that the transposition events might lead to genome instability. ERVs and other family members of TEs used to be considered as “junk DNA,” but with the technological advancement in genome-wide expression and epigenetic profiling, we started to appreciate more on their functional contribution to development and diseases. We now understand that the complexity of the mammalian genome is not achieved through a significant increase of the protein-coding sequences, but by the vast expansion of regulatory capacities imparted by the non-coding sequences. TEs occupy nearly half of the non-coding genome and thus are thought to play critical roles in shaping the complexity of mammalian gene regulatory network.

Comparing to other repeat element families, such as short interspersed nuclear elements (SINEs) and long interspersed nuclear elements (LINEs), ERVs bear more sequence complexities and thus may play more specific regulatory functions in the genome [1, 3]. Although ERVs are the smallest class of retrotransposon family, they exhibit significant enrichment and are over-represented in cell type-specific active regulatory sequences [4]. ERVs are thought to be generated as by-products of retroviral infection and integration events in the ancestral mammalian genome. During the evolution, they were endogenized and inherited through germline transmission [5]. Most ERVs are tamed now in the host genome through mutations of their transposition machinery or through coevolution of host regulatory factors that repress ERV activation [5]. A full-length ERV consists of two long-terminal repeats (LTRs) flanking at both 5′ and 3′ sides, and the open reading frame (*GAG*, *POL*, and *ENV*) in the center. It should be emphasized that LTRs are the regulatory elements of ERVs [6]. The LTR regions of ERVs possess binding sites for a broad scope of transcription factors to interact with the host gene regulatory machinery and achieve precise control of ERV activity [7]. Meanwhile, exaptation of the LTRs' cis-regulatory functions (enhancer and promoter) also leads to innovations of the transcription network in the host genome. ERVs also exclusively possess the primer binding site (PBS) which can recruit complementary tRNA to prime for viral reverse transcription. PBS sequences are also found to be the binding sites for ERV silencing factors from the host [7]. Based on the similarity to tRNA sequence in the PBS region, ERVs can be further classified into several families, ERVH, ERVW, ERVK, ERVL, etc. Out of the 8% genomic constitution of ERVs in human genome, 90% exist as solitary ERVs with only the LTR sequences present and the viral protein-coding ERV-int regions shed off [3].

**Figure 1:**
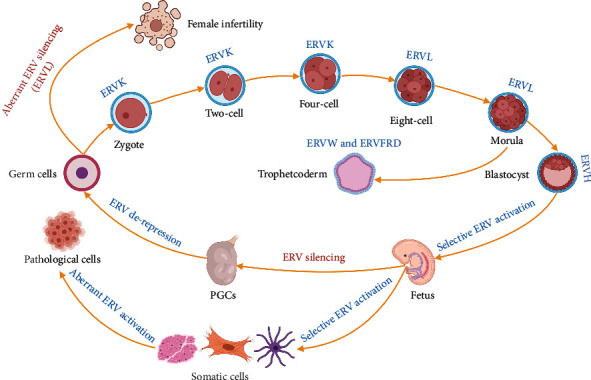
The dynamic regulation ERVs during development. During embryonic and somatic development, ERVs are selectively activated, whereas aberrant activation or silencing of ERVs results in pathological consequences.

The expression level of ERVs is dynamically regulated in early embryogenesis, differentiated tissues, and germ cells [8]. Interestingly, the expression of different ERV sub-families exhibited high temporal specificity during early human embryo development [8], suggesting ERVs as stringent markers for specific embryonic stages (Figure 1). Besides, many shreds of evidence also demonstrated that abnormal ERV expression may lead to different types of diseases [9–13]. ERVs can affect genome-wide transcription through multiple layers of regulation as discussed below. Thus, their activities should be tightly controlled in the mammalian genome to coordinate with proper development and cell fate decision process. The precise control of ERVs in the host genome is largely through transcriptional and epigenetic regulation. DNA methylation is considered a common regulatory mechanism to repress ERV expression. Many ERVs in human are heavily methylated and silenced in differentiated tissues but show loss of methylation and aberrant expression in cancer [14]. Apart from DNA methylation, the Krüppel-associated box domain-containing zinc finger protein (KRAB-ZFP) is known to regulate chromatin configuration surrounding ERV elements [15]. ERV elements are bound by zinc finger domains of the KRAB-ZFPs, and the KRAB domain can recruit tripartite motif-containing 28 (TRIM28), resulting in the trimethylation of histone H3 lysine9 (H3K9me3) and ERV silencing in embryonic stem cells [16]. Histone deacetylation is also involved in ERV regulation. It has been found that histone deacetylase inhibitor (HDACi) treatment led to ERV9 activation which prevented testicular cancer progression, but this did not lead to upregulation of other ERV sub-families, implying that histone deacetylation may regulate human ERV silencing in a sub-family-specific manner [15, 17]. In general, it can be envisaged that a combination of different kinds of epigenetic modifications is orchestrated to tightly control the ERV activity.

Over the last 10 years, increasing pieces of evidence are showing that LTRs may play under-recognized regulatory roles in mammalian development and diseases [9–13]. In the following sessions, we will discuss in detail about the current knowledge on the functions of ERV in chromatin and transcription regulation, how these functions are achieved, and how they contribute to cell fate decision during mammalian embryonic development and stem cell differentiation.

## 2. The Functions of ERV in Gene Regulation

If chromatin regulation is a symphony, then ERV has several instruments to play. ERV recruits transcription factors, works as alternative promoters, encodes long non-coding RNAs (lncRNAs), and produces protein products to mediate cellular function. These abilities could be stemmed from intrinsic functions of ERV or could be coopted during the coevolution with the host genome. Nevertheless, the functions of ERV have become an integral part of the regulatory machinery in the genome and indispensable for the normal development and homeostasis of mammals.

### 2.1. The Recruitment of Transcription Factors

In-silico mapping revealed that many ERVs are enriched with transcription factor-binding sites, suggesting ERVs may act as cis-regulatory elements for transcription [4]. Putative epigenetic markers for promoter and enhancer, such as H3K4me3 and H3K27ac, are frequently seen on the LTR regions [11]. Activated ERVs are largely associated with cell type-specific open chromatin configuration. For example, in human pluripotent stem cell, HERVH sub-family is enriched with binding sites for pluripotency transcription factors such as OCT4 and KLF4, as well as active histone modifications like H3K4me3 and H3K27ac, adopting open chromatin conformation [11, 18]. In addition, DUX4, as well as its mouse homologous DUX, can bind to the ERVL sub-family in human and mouse, respectively. This leads to epigenetic activation of genes downstream of the ERVL elements, which are essential for initiating zygote genome activation (ZGA) in early human and mouse embryos [19]. Human DUX4 is kept silenced in differentiated tissues, as aberrant activation of DUX4 in muscle tissue upregulates HERVL, leading to unscheduled transcription activation of early embryonic genes which eventually resulted in facioscapulohumeral muscular dystrophy [10]. These shreds of evidence together suggested that ERVs can recruit transcription factors to actively influence the epigenetic landscape in the nearby region, thus contributing to cell type-specific gene regulation.

Moreover, ERVs can also modulate signaling pathways to coordinate cell fate change. It has been found that ERVs shaped the evolution of the transcription network underlying the interferon response [20]. For instance, one of the ERV sub-families, MER41, is enriched with interferon-induced STAT1-binding sites [20]. STAT1-bound MER41 regions were enriched with H3K27ac upon interferon stimulation. The knockout of MER41 impaired the expression of interferon-induced genes such as *AIM2* which senses cytosolic foreign DNA and activates inflammatory responses [20]. This suggests that ERV can sense the interferon signaling pathway and feedback to regulate innate immunity.

### 2.2. Alternative Promoters and Alternative Splicing

The LTR elements in ERVs possess the intrinsic promoter activity to drive ERVs expression. LTRs can also function as alternative promoters to drive host ORF expression. It has been estimated that up to 75% of human genes take advantage of alternative promoters to achieve tissue-specific regulation [21]. The employment of ERVs as alternative promoters not only results in stage- or tissue-specific gene expression patterns but also generates different isoforms of proteins [3, 21, 22]. Besides, ERVs are found over-represented in regions close to protein-coding sequences, suggesting that they are closely related to transcription initiation in the genome [23]. For instance, MT2 of the mouse ERVL sub-family is highly activated in mouse 2C embryo and functions as an alternative promoter to upregulate MERVL nearby genes, generating chimeric transcripts with junctions to MERVL elements [24]. An example to demonstrate is that *Zfp352* has two promoters (P1 and P2) that are active in mouse early embryo and somatic cells, respectively [25–27]. Interestingly, the active promoter of *Zfp352* in early embryos overlaps with MT2B1 repeats, indicating the ERV promoter may be critical for the early activation of *Zfp352* [25–27]. A recent large-scale transcriptomic analysis discovered that 23% of all protein-coding genes expressed in various cancer types possess at least two promoters that cause a significant tumor type-specific change in isoform expression [28]. For example, *JAZF1* prefers the 3′ full-length promoter (prmtr.40310) in KIRP cancer, whereas in KIRC cancer, a truncated promoter (prmtr.40312) is favored [28].

The presence of alternative promoters not only leads to context-dependent gene activation but also creates alternative splicing variants of the transcripts [21]. Alternative splicing can occur in the retroviral RNA itself, which has been correlated to cancer initiation[9]. For example, the open reading frame of HERVK provides a source for alternative splicing, and the spliced variants of HERVK can be detected in various cancers, some of which are cancer type-specific [29]. The differentially expressed retroviral RNA isoforms raise questions of how these isoforms are generated, and what functional differences exist between these isoforms. Apart from retroviral isoforms, ERVs are also involved in generating alternatively spliced isoforms in coding genes. For instance, the upstream MER4A can be utilized as an alternative promoter for *GTSO1*, which led to the generation of 15 isoforms of GTSO1 that may function differently under different disease contexts [30].

### 2.3. ERV-Derived Long Non-coding RNA

More importantly, many ERVs can encode for lncRNA. The functions of these lncRNAs can be involved in various processes like recruiting transcription factors, cooperating with epigenetic regulators or modifiers, or interacting with miRNAs [31–33].

A few studies demonstrated that the ERV-derived lncRNAs can participate in signaling transduction by regulating protein recruitment and protein degradation [34–37]. One of the ERV sub-family members, ALVE1, transcribes into lnc-ALVE1-AS1 to activate the TLR3 signaling pathway in the cytoplasm and induce antiviral innate immunity [35]. In addition, transcriptome analysis revealed that a human ERV-derived lncRNA, termed TROJAN, binds to metastasis-repressing factors and promotes their degradation through ubiquitin-associated signaling pathway [36], thus promoting breast cancer progression. On the converse, antisense oligonucleotide repressing TROJAN slows down the breast cancer progression extraordinarily *in vivo*, suggesting that TROJAN promotes cancer invasion and can serve as a potential therapeutic target [36].

### 2.4. ERV-Derived Proteins

In addition to RNAs, the proteins translated from ERVs can also perform specific functions under certain contexts. These proteins are derived from the open reading frame of ERV, including *GAG*, *POL*, and *ENV*. The functions of these viral proteins are diversified [38–40]. For instance, the ENV protein from HERVK can upregulate the p-ERK1/2 and RAS signaling pathways in human pancreatic cancer, and knockdown of ENV suppressed the activity of the ERK signaling pathway [40]. Moreover, ENV proteins from HERVW and HERVFRD aid in trophectoderm cell fusion and facilitate mammalian embryo implantation into the uterus [41, 42], and the GAG protein produced by HERVK promotes prostate cancer progression by inducing androgen hormone release [38].

## 3. ERV in Stem Cell Differentiation

Embryonic development is initiated after fertilization, followed by zygote cleavage. In the early embryo cleavage stages, the zygotic genome is activated, accompanied by global remodeling and rewiring of the transcription network. Before the first cell fate segregation in late morula and blastocyst, cells in embryos retain the capacity to give rise to the complete embryo proper and are thus considered totipotent. In blastocyst, cells are committed to the outer layer trophectoderm and inner cell mass which gives rise to the pluripotent epiblast and differentiates into three germ layers and somatic tissues. Numerous genetic and epigenetic programs governing the embryo developmental processes have been revealed, but mostly focusing on the regulation of the coding genome. Non-coding elements such as ERVs are poorly understood in this context but are increasingly gaining attention. ERVs are extensively activated in early embryo development, with a highly restricted spatial-temporal pattern, and are drastically silenced during differentiation with exceptions of extraembryonic tissue and germlines (Figure 1). Here, we will focus on the functions and regulation of ERVs in a few key developmental stages and context to discuss the emergent roles of ERVs in chromatin regulation and stem cell differentiation.

### 3.1. ERV in Totipotency Regulation

During both mouse and human embryo development, ERVL subfamily is activated around ZGA but gradually silenced thereafter. It seems that ERVL is predominantly associated with the totipotent state. In mouse, transcripts from MERVL loci occupy 2% of the total mRNA in 2C embryo [24]. More than 307 genes were found to form chimeric transcripts with partial MERVL sequence [24]. These chimeric transcripts are mostly associated with metabolism and transcription regulation involved in mouse ZGA. For instance, in mouse 2C embryo, MT2-SPIN chimeric transcript excludes 3 exons at the N-terminus compared to the native isoform [43], resulting in the native and chimeric isoforms of SPIN that bear different phosphorylation sites by MAPK [43] and thus may mediate different signaling functions. MT2, together with partial MERVL-int sequence, is also a robust fluorescence reporter for 2C embryo as well as 2C-like cells in mouse embryonic stem cells (mESCs) [24]. MT2 also exhibits regulatory functions in activating distal 2C-specific genes. MT2 drives *Zscan4* cluster gene expression in mouse 2C embryo, and the upregulation of *Zscan4* can further activate MT2, resulting in DNA demethylation and open chromatin configuration to further activate 2C-specific genes nearby MT2 loci [44]. Interestingly, ectopic activation of MERVL by CRISPR activation system also resulted in the upregulation of 2C genes [45], implying that MERVL can act as a cis-regulatory element to control totipotent gene expression.

Similarly, in human, HERVL expression is also enriched in 8C stage corresponding to the time of human embryo ZGA [8]. MERVL and HERVL can be bound by mouse DUX and human DUX4, respectively, but cross-species binding is minimum, suggesting independent but converged evolution in mouse and human [19, 46]. Over-expression of *Dux* in mESCs can activate MERVL and downstream 2C genes. Similarly, human *DUX4* over-expression results in HERVL activation and simultaneously upregulation of human 8C-specific genes [19, 46].

Upon exiting from 2C stage, MERVL is rapidly silenced and its expression falls back to baseline in mouse 8C embryos. The silencing of MERVL is mediated by ZFP809. ZFP809 is a mouse-specific zinc finger protein, containing the KRAB domain at the N-terminus and seven zinc finger domains at the C-terminus [47]. The zinc finger domains allow ZFP809 to bind to the PBS sequence of MERVL, and the KRAB domain recruits TRIM28, together with NURD (histone deacetylase) and SETDB1 (histone methyltransferase), which led to condensed chromatin configuration and repression of MERVL activity [7, 47, 48]. Interestingly, it is noted that *Zfp809* produces two isoforms: a full-length protein and a truncated protein that lacks 50 residues at C-terminus. The full-length protein is selectively stable in ESCs but degraded in other cell types. Whereas the short isoform is constitutively expressed in both ESCs and differentiated cells, but the underlying impact and functional differences between the two differentially expressed isoforms remain unknown [47]. Nevertheless, a critical question that remained to be validated is whether the failure to silence MERVL will lead to the delay in the development of mouse early embryos, trapping the cells in totipotency.

### 3.2. ERV in Pluripotency Regulation

Upon exiting from totipotent state, cells take on the first cell fate decision to become extraembryonic trophectoderm or pluripotent epiblast. ERVL is rapidly silenced along with the exit from totipotency, while other sub-families of ERVs are upregulated [8, 11, 45]. HERVH sub-family is one of the most predominant ERVs in pluripotent stem cells. The internal sequence (ERV-int) is degenerated in a slower manner compared to other ERVs, suggesting the potential function of HERVH-int sequence in the pluripotent state [5, 6]. It is not known whether the silencing of HERVL is a prerequisite for the activation of HERVH during human embryo development. But it is possible that if HERVL is not silenced, the totipotency transcription network will remain active, and cells might be trapped in the totipotent state. Similarly, forced activation of HERVL in pluripotent stem cells may also induce totipotent gene expression and shut down HERVH expression [45].

HERVH copies are highly enriched with the putative binding sites for pluripotent factors including KLF4, NANOG, and OCT4 [11]. In hESCs, HERVH is also enriched with H3K4me3 and H3K27ac [11], implying that they are potentially active promoters or enhancers for pluripotent gene regulation. Ectopic expression of HERVH sub-families by CRISPR activation system can result in an extensive upregulation of genes up to 200 kb nearby of HERVH sequences [49]. Besides, a total of 128 and 145 chimeric transcripts of HERVH are detected in hiPSCs and hESCs respectively, suggesting HERVH can function as alternative promoters to activate pluripotency-related genes [11]. In contrast, native promoters of these genes are rarely active in pluripotent stem cells [11]. Although there could be potential functional distinctions between chimeric transcripts from ERV promoters and original transcripts from native promoters, the ERV-mediated activation of these genes in early embryonic development offers additional opportunity to rewire gene expression and innovate on the transcription regulation.

In addition, the lncRNAs derived from HERVH also play critical roles in pluripotency regulation. They may function as scaffold units to recruit chromatin modifiers and direct them towards specific locations [50, 51]. In detail, the HERVH lncRNAs mainly localize to the nucleus, and they can recruit chromatin modifiers such as P300 to the genomic loci of LTRs to regulate transcription of pluripotency genes nearby [52]. HERVH knockdown leads to fibroblast-like cell morphology [52] and downregulates more than 1000 genes observed, including a 50% reduction in NANOG and OCT4 expression, resulting in the partial loss of pluripotency and upregulation of differentiation markers [11]. In line with its role in hESCs, HERVH exhibited similar functions during somatic cell reprogramming [52]. HERVH expression is substantially upregulated upon ectopic expression of reprogramming factors, while depletion of HERVH during reprogramming leads to a reduction of iPSC colony-forming efficiency [52]. These shreds of evidence together indicate that HERVH is indispensable for both pluripotency establishment and maintenance.

Despite the importance of HERVH, pieces of evidence have been controversial about whether HERVH is required for naïve or primed pluripotency [11, 36, 52, 53]. Based on the LTR regions, HERVH can be further divided into several sub-families, such as LTR7Y, LTR7B, and LTR7. Some of the LTRs, like LTR7, are predominantly expressed in primed pluripotency [8], while LTR7Y may be more specific to naïve pluripotency [8]. Thus, naïve and primed pluripotency might employ different sub-families of HERVH controlled by the respective LTRs, but how this specificity is achieved requires further investigation.

### 3.3. ERV in Extraembryonic Tissue Differentiation

Research work has shed more light on the roles of ERVs in trophectoderm differentiation since the 1990s [54]. The roles of ERVs in extraembryonic tissue differentiation are mediated by regulating trophectoderm-specific transcription and by encoding for fusion proteins during the syncytia formation.

Many ERVs have a robust expression in placenta development [55]. Among all, HERVW, HERVFRD, and HERV3 are the top three active sub-families that encode for a high level of *ENV* gene [56, 57]. The SYNCITIN 1 translated from *the ENV* gene of HERVW lacks an immunosuppressive domain compared to full-length ENV protein. It is specifically upregulated in syncytiotrophoblast during implantation [41, 42]. The hydrophobic domain in SYNCITIN 1 enables its fusion with plasma membrane and potentially aids in uterus invasion [58]. Ectopic expression of HERVW *ENV* gene can induce cell fusion, which is reversed by neutralizing antibodies against SYNCITIN 1 [59]. In contrast, the lack of SYNCITIN 1 in primary trophoblast cells reduces the ability to form syncytia [60]. Similar to SYNCITIN 1, the SYNCITIN 2 produced by HERVFRD also promotes cell fusion upon ectopic expression in several cell lines [61]. Interestingly, ENV protein derived from HERV3 is expressed not only in syncytiotrophoblast but also in a wide range of tissues, particularly those producing hormones [62, 63]. More importantly, 1% of the Caucasian population bears a premature stop codon near the N-terminus, resulting in a non-functional short isoform of the protein. However, this does not lead to observable physiological defects in these individuals [54, 55]. Different ENV proteins from different ERV sub-families might play redundant roles. Apart from the proteins, ERVs also function as cis-regulatory elements in extraembryonic differentiation. In the mouse placenta, one of the ERV sub-families, RLTR13D5, is highly enriched with H3K27ac and H3K4me1, suggesting its potential role as an enhancer [64]. Moreover, RLTR13D5 can be functionally bound by CDX2, EOMES, and ELF5 to regulate the transcription in trophoblast stem cells and contribute to placenta development [64].

### 3.4. ERV in Somatic Tissue Differentiation

Despite the high activity of ERV and other TE families in early embryos, they are thought to be largely deactivated during the differentiation process. The silencing mechanism involves coevolution between the host transcription regulatory machinery and ERVs to tame ERV expression and limit their transposition. Improper silencing of ERVs is associated with loss of tissue homeostasis and pathological conditions. For example, the ENV protein derived from HERVW is highly expressed in type-1 diabetes and inhibited the secretion of insulin [65]. Transcripts and proteins of HERVK are also detected in amyotrophic lateral sclerosis brain tissue, which may contribute to the inhibition of neurite growth [66]. In human muscle cells, aberrantly expressed DUX4 binds to and induces HERVL expression, which serves as alternative promoters to alter the transcription network in facioscapulohumeral muscular dystrophy [10]. Moreover, HERV-derived lncRNA TROJAN promotes ubiquitin-associated degradation of metastasis-repressing factors and accelerates breast cancer progression [36].

In addition to the conventional view that ERV activation in differentiated tissue led to pathological conditions, more and more tissue-specific ERVs were identified, and they are thought to contribute to the cell type-specific differentiation or tissue-specific functions [67]. For instance, during mouse gastrulation, different ERV sub-families were activated in various cell fates: erythroid has high RLTR10F activity, while mesoderm favors ERVB4 [67]. However, the exact function of these ERVs in the respective lineage remains elusive. Similarly, during the differentiation of human pluripotent stem cells (hPSCs) to cardiomyocytes *in vitro*, distinct sets of ERVs are selectively activated in different cell populations. For example, LTR32, MER57A-int, and MER45A are specifically expressed in definitive cardiomyocytes while MLT1H1, HERVIP10B-int, and LTR5A are selectively active in non-contractile cells [67]. It is noted that many ERV transcription regulators in ESCs, such as KLF-family members, are also expressed in tissue-specific cell types; thus, they may regulate ERV in the respective context.

Taken together, these pieces of evidence demonstrate two fundamental aspects of ERV in differentiation: (1) Various ERVs are now associated with tissue differentiation and specific cell lineage. (2) Aberrant ERV expression in differentiated tissues may be toxic while targeting these ERVs could provide potential therapeutic means to slow down disease progression.

### 3.5. ERV in Germline Formation

Although ERV activity may be largely silenced during differentiation, it is highly expressed and activated during germline formation. The first observation of ERV expression in germline cells can be dated back to 1983 when virus-like “intracisternal A particle (IAP)” was detected in mouse oocytes [68]. Up to now, more than 800 types of LTRs are detected in mouse oocyte, and they are involved in diverse functions, which aid in oocyte transcription regulation and facilitate oogenesis [69]. For instance, DICER protein is present in both mouse somatic cells and oocytes [70]. However, instead of being transcribed from native promoters, oocyte-specific DICER expression is driven by the LTR of MTC and produces an isoform lacking the N-terminal DExD helicase domain compared to full-length somatic DICER produced by its native promoter. And the deletion of LTR regions of MTC impaired oocyte-specific DICER, resulting in female sterility [70]. Many of the activated ERVs in the oocyte are passed down to the zygote as maternal factors, which are thought to be involved in ZGA [71], but their exact functions remain to be dissected in the future.

On the contrary, the progenitors of mouse germline cells, namely primordial germ cells (PGCs), show repressed ERV activity. ERV sequences are enriched with H3K9me3 and H3K27me3 that induce a repressive chromatin configuration [72]. In detail, SETDB1 as a methyltransferase protects PGCs from ERV activity. SETDB1 knockout PGCs show upregulated ERV activity, low survival rate, and postnatal hypogonadism [72]. Although this is in contrast to the general knowledge that ERVs are upregulated in germ cells, it is possible that different families of ERVs are involved in various stages of germ cell formation.

## 4. Conclusion and Outlook

ERVs are previously thought to coordinate with the host genome during mammalian evolution, and now they are considered as integral parts to form species and cell type-specific gene regulatory networks. The research of ERVs in stem cell fate decision and differentiation has just been unraveled, and many questions remained to be answered. Given the observed stage-specific expression pattern of ERV (Figure 1), what will be the specific function of each ERV sub-family in different developmental stages? How do different cell types achieve specific activation of ERV sub-families? What is the consequence of unscheduled activation or silencing of ERVs during early embryogenesis? Are ERVs exhibiting cell type-specific expression beyond blastocyst stages? Will ERV represent novel targets for diseases? Future studies will shed light on these questions and open up the fascinating but less charted road of ERVs.
